# PP75 Treatment Of Rheumatoid Arthritis In The Single Health System: Profile Of Medication Use And Expenses

**DOI:** 10.1017/S0266462324002447

**Published:** 2025-01-07

**Authors:** Adson José Moreira, Wallace Mateus Prata, Francisco de Assis Acurcio, Augusto Guerra, Juliana Alvares-Teodoro

## Abstract

**Introduction:**

Rheumatoid arthritis (RA) is a chronic inflammatory disease. Treatment aims to improve functional capacity and quality of life. Biological disease-modifying antirheumatic drugs (DMARDs) and oral inhibitor of Janus kinases (JAKs) used in treatment are high cost. The objective of this study was to analyze the profile of use and expenses of these medicines used in Brazil’s Unified Health System (SUS).

**Methods:**

This is a retrospective cohort of users who received at least one biological DMARDs or oral inhibitor of Janus kinases (JAKs) registered in the Open Room on Health Intelligence—Sabeis/Datasus. The valuation of the 11 drugs standardized by the Clinical Protocols and Therapeutic Guidelines (PCDT) was carried out based on public purchases registered in the Price Bank in Health and an active search with the Ministry of Health, between 2017 and 2021. Expenses were calculated based on the number of High Complexity Procedure Authorizations (APACs) registered in the SIA/SUS database.

**Results:**

Evaluation of 195,163 patient dispensing records was undertaken. Spending on medicines was BRL408,146,527 (USD79,012,759) in 2017, BRL237,615,290 (USD46,002,748) in 2018, BRL213,790,222 (USD41,350,815) in 2019, BRL195,626,575 (USD37,837,644) in 2020, and BRL140,096,142 (USD27,109,717) in 2021. It was found that in 2017, adalimumab (33%), etanercept (28%), and golimumab (14%) were the most used biological DMARDs. It is worth noting that in 2017, adalimumab and etanercept represented more than 50 percent of the costs of treating RA in the SUS. In 2021, it was found that 61 percent of total expenses were distributed between the drugs etanercept (23%), golimumab (18%), certolizumab (14%), adalimumab (11%), and tofacitinib (11%).

**Conclusions:**

In the period from 2017 to 2021, changes can be observed in the profile of use and expenditure of biological DMARDs and oral inhibitor JAKs, resulting from the incorporation of new drugs and the entry of biosimilars in the Brazilian market. However, adalimumab, one of the first biological DMARDs incorporated, is the most consumed and least expensive medicine.

